# Cardiac magnetic resonance haemodynamics in paediatric heart transplant patients: fick oximetry versus cardiac magnetic resonance phase contrast

**DOI:** 10.1017/S1047951123001440

**Published:** 2023-06-15

**Authors:** Jennifer Schramm, Ileen Cronin, Robert McCarter, Jason G. Mandell, Tacy Downing, Joshua Kanter, Russell Cross, Laura Olivieri

**Affiliations:** 1 Department of Anesthesia and Critical Care Medicine, Johns Hopkins University, Baltimore, MD, USA;; 2 Department of Pediatric Cardiology, Seattle Children’s Hospital, Seattle, WA, USA;; 3 Children’s National Medical Center, Washington, DC, USA;; 4 Division of Pediatric Cardiology, University of Rochester Medical Center, Rochester, NY, USA;; 5 Department of Pediatric Cardiology, Children’s National Medical Center, Washington, DC, USA; 6 Department of Pediatric Cardiology, Children’s Hospital of Pittsburgh, One Children’s Hospital DrivePittsburgh, PA, USA

**Keywords:** Interventional cardiac magnetic resonance, pediatric heart transplantation, radiation safety

## Abstract

**Background:**

Lifetime radiation exposure for paediatric orthotopic heart transplant (OHT) patients is significant with cardiac catheterisation as the dominant source. Interventional cardiac magnetic resonance is utilised to obtain simultaneous, radiation-free haemodynamics and flow/function measurements. We sought to compare invasive haemodynamic measurements and radiation exposure in traditional cardiac catheterisation, to comprehensive interventional cardiac magnetic resonance.

**Methods:**

Twenty-eight OHT patients who underwent 67 interventional cardiac magnetic resonance procedures at Children’s National Hospital were identified. Both invasive oximetry with peripheral oxygen saturation (Fick) and cardiac magnetic resonance phase contrast measurements of pulmonary and systemic blood flow were performed. Systemic and pulmonary blood flow from the two modalities was compared using Bland–Altman, concordance analysis, and inter-reader correlation. A mixed model was implemented to account for confounding variables and repeat encounters. Radiation dosage data were collected for a contemporaneous cohort of orthotopic heart transplant patients undergoing standard, X-ray-guided catheterisation.

**Results:**

Simultaneous cardiac magnetic resonance and Fick have poor agreement in our study based on Lin’s correlation coefficient of 0.68 and 0.73 for pulmonary and systemic blood flow, respectively. Bland–Altman analysis demonstrated a consistent over estimation of cardiac magnetic resonance cardiac output by Fick. The average indexed dose area product for patients undergoing haemodynamics with endomyocardial biopsy was 0.73 (SD ±0.6) Gy*m^2^/kg. With coronary angiography added, the indexed dose area product was 14.6 (SD ± 7.8) Gy*m^2^/kg.

**Conclusions:**

Cardiac magnetic resonancemeasurements of cardiac output/index in paediatric orthotopic heart transplant patients have poor concordance with Fick estimates; however, cardiac magnetic resonance has good internal validity and inter-reader reliability. Radiation doses are small for haemodynamics with biopsy and increase exponentially with angiography, identifying a new target for cardiac magnetic resonance imaging.

Paediatric orthotopic heart transplant patients experience significant medical radiation exposure due to cardiac and non-cardiac imaging required pre- and post-transplant.^1,2^ In a small cohort study, the mean cumulative effective dose is 53.5 millisieverts (~1000 chest X-rays) in the first year after transplant; more than half is associated with cardiac catheterisation.^1^ Previous work by Congenital Cardiac Catheterization Outcomes Project-Quality Improvement Initiative showed the median dose for haemodynamics plus biopsy alone was 5 Gy*m^2^/kg (IQR 3–11 Gy*m^2^/kg), and for complete annual catheterisation with coronary angiography was 46 Gy*m^2^/kg (IQR 29–74 Gy*m^2^/kg).^3^ This study was done in 2019, however, with older versions of fluoroscopic equipment. Therefore, it is important to examine strategies that reduce radiation exposure in this population while maintaining the ability to monitor haemodynamics using non-fluoroscopic techniques such as cardiac magnetic resonance.^4,5^

Cardiac magnetic resonance is increasingly used as a radiation-free way to identify rejection in cardiac transplant patients with multiple modalities undergoing investigation.^6–8^ Cardiac magnetic resonance can be used to characterise tissue oedema, scar, perfusion defects, and quantify function more accurately than echocardiography alone.^7,9–11^ However, cardiac magnetic resonance measurements of cardiac output have not been compared to direct catheter-based haemodynamic measurements in paediatric orthotopic heart transplant patients nor has there been analysis of inter-reader reliability of cardiac magnetic resonance measurements in this population.

Interventional cardiac magnetic resonance can collect a wealth of anatomic and physiologic data under one anaesthesia. The first description of interventional cardiac magnetic resonance is from 2004 with a group of adult and paediatric patients. The cardiac magnetic resonance data were collected simultaneously with invasive haemodynamics with good agreement.^12^ The largest interventional cardiac magnetic resonance feasibility study in children came in 2017 in 50 children and was followed in the same year by a larger study in 102 adults demonstrating good agreement between the two modalities.^13,14^ Follow-up studies have confirmed its utility in various physiologic states.^15^ In certain patient populations, however, the specific anatomy and/or physiology may lead to significant disagreement between the two modalities.^16,17^ Some of the studies also had sequential sampling; cardiac magnetic resonance first and then haemodynamics. The studies all varied in their anaesthetic technique and patient sizes, known to vastly affect haemodynamics.

The aim of this study is to compare cardiac output measurements from traditional cardiac catheterisation with fluoroscopic guidance to interventional cardiac magnetic resonance within a paediatric orthotopic heart transplant cohort to evaluate concordance between the modalities. The second aim is to measure radiation doses from fluoroscopic catheterisation on up-to-date equipment to show what would be avoided with interventional cardiac magnetic resonance.

## Materials and methods

### Prospective interventional cardiac magnetic resonance

In an institutional review board approved study, with consent/assent as required, paediatric heart transplant patients of any age referred for medically necessary cardiac catheteriaation underwent magentic resonance-guided right heart catheterisation under general anaesthesia in the interventional cardiac magnetic resonance suite at Children’s National, based on standard methods previously described.^13^ Imaging was completed using a 1.5 T Aera (Siemens, Erlangen, German), including standard volumetry and flow measurements, with parameters tailored to patient size according to guidelines and lab standards.^13,18,19^ Baseline interventional cardiac magnetic resonance imaging was followed by cardiac catheterisation in the interventional cardiac magnetic resonance suite using standard real-time imaging guidance to collect oximetry and pressure data.^13^ Heart rates at the time of pulmonary artery sampling (which served as the mixed venous saturation) were used to find the assumed oxygen consumption.^20^ The interventional cardiac magnetic resonance lab was not equipped at the time of this study to perform thermodilution and the ventilator modules for measuring oxygen consumption are not interventional cardiac magnetic resonance compatible, thus the assumed oxygen consumption was used. Pulmonary vein saturations were assumed to be equal to systemic saturations as measured by either pulse oximetry or invasive arterial monitoring when available. The Fick equation was then used to calculate pulmonary and systemic blood flow. Cardiac volumes, flow rates in the ascending aorta and main pulmonary artery were measured using offline software (Medis Medical Imaging, Leiden, the Netherlands). Flow measurements were performed by cardiologists with expertise in cardiac magnetic resonance and interrater reliability was assessed by a blinded observer who measured flows in all cases.

### Controls for radiation measurement

Contemporaneous heart transplant patients who underwent standard fluoroscopy-guided catheterisation between 2018 and 2020 provided data for radiation dose estimation. Single plane fluoroscopy and angiography procedures were performed in a single catheterisation suite with up-to-date equipment by two providers (Siemens Artis Q biplane and Zen biplane with large- and medium-sized detectors, respectively). Patients were excluded if they were critically ill (resulting in elevated procedure times) or had additional procedures requiring fluoroscopy outside of biopsy or coronary angiography. The demographics and total indexed dose area product were collected and then converted to Gy*m^2^/kg. Patients were then stratified into two groups: (1) follow-up for rejection with surveillance haemodynamics and (2) traditional complete annual catheterisation. Patients in the follow-up for rejection group had haemodynamics and endomyocardial biopsy under fluoroscopy. Complete annual catheterisation patients had haemodynamics, endomyocardial biopsy, and coronary angiography under fluoroscopy. A group with haemodynamics alone could not be retrospectively created with the current fluoroscopy equipment limiting full direct comparison.

### Analysis

We used the Kolmogorov–Smirnov test to evaluate the normality assumption prior to conducting Bland–Altman and concordance analysis to compare cardiac magnetic resonance- and Fick-generated measurements of pulmonary and systemic blood flow using Stata (StataCorp. 2019. *Stata Statistical Software: Release 16*. College Station, TX: StataCorp, LLC). We also evaluated the correlation between differences in haemodynamic measurements from the same patients to determine whether repeat measurements could be treated as independent prior to Bland–Altman and concordance analysis. In these analyses, systemic blood flow was defined as total caval return when comparing cardiac magnetic resonance and Fick Qp. Pulmonary blood flow was defined as pulmonary venous return when comparing cardiac magnetic resonance and Fick Qs. These definitions were assigned recognising that aortopulmonary collateral flow may be present in those with single ventricle physiology prior to orthotopic heart transplant. In a subset of 67 patients with no significant valvular regurgitation, the concordance of cardiac magnetic resonance measures of pulmonary blood flow and systemic blood flow and right and left ventricular cardiac output was evaluated using Lin’s correlation coefficient.

Once Bland–Altman and concordance analyses were completed, a mixed-effects model was implemented to control for multiple clinical and demographic variables when evaluating paired differences between cardiac magnetic resonance and Fick measurements. Finally, interrater reliability for flow measurements was assessed using a two-tailed intraclass correlation coefficient.

## Results

### Hemodynamic agreement

**Figure 1.**
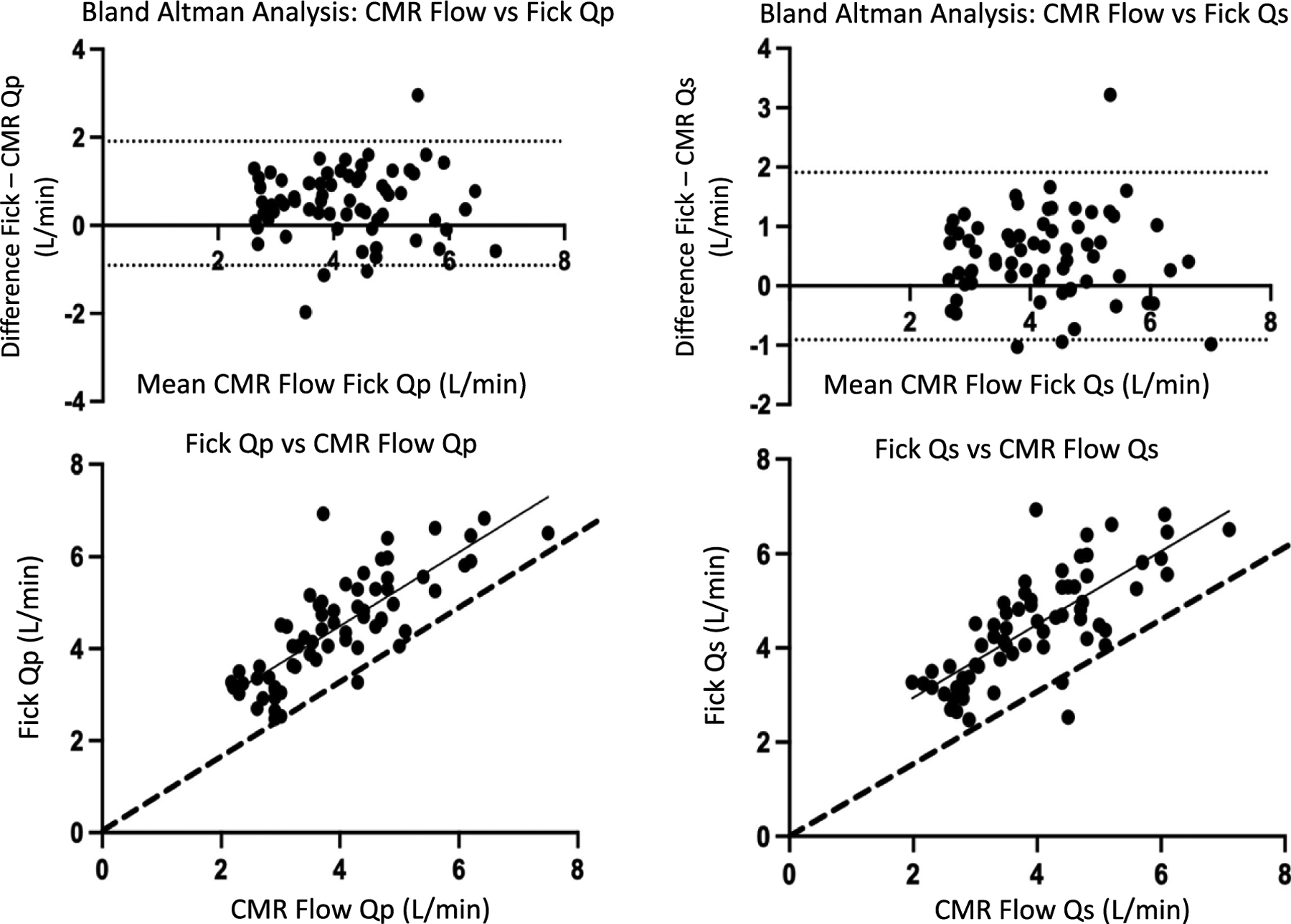
***ad***: bland-altman and correlation plots. panels a and C represent comparisons for systemic blood flow (systemic venous return), ***b*** and ***d*** represent those for pulmonary blood flow (pulmonary venous return). 2***a*** and 2***b*** represent the bland-altman analysis for CMR flows versus fick. dots represent individual measurements. The dotted lines represent the 95% limits of agreement. 2***c*** and 2***d*** represent the correlation plots for fick versus CMR flows. the solid line is the best fit line with the dotted line representing perfect agreement.

Sixty-seven interventional cardiac magnetic resonance procedures in 28 unique heart transplant patients with usable cardiac magnetic resonance and catheterisation data were included in analysis (Table 1). Two studies were excluded due to inaccurate cardiac magnetic resonance triggering and image artifacts that compromised accuracy. The interventional cardiac magnetic resonance versus Fick group tended to be younger at time of transplant, but had longer time from transplant to procedure indicative of well-established grafts. Patients who had haemodynamics and endomyocardial biopsy alone tended to be newer transplants with multiple catheterisations performed in the first year. After validating the normality assumption and negligible effect of repeat assessments, Bland–Altman analysis for comparison of cardiac magnetic resonance and Fick measurements of cardiac had Lin correlation coefficients 0.68 and 0.73 for pulmonary blood flow and systemic blood flow, respectively. The bias for both datasets was + 0.5 L/min with 95% limits of agreement of −1.0 – + 2.1 for pulmonary blood flow and −0.9 – + 1.9 for systemic blood flow (Fig. 1, panels A, B). This indicates Fick consistently produces estimates of cardiac output that exceed cardiac magnetic resonance estimates by an average of 0.5 L/min. The R^2^ for both datasets was 0.6 (Fig. 1, panels C,D).

**Figure 2.**
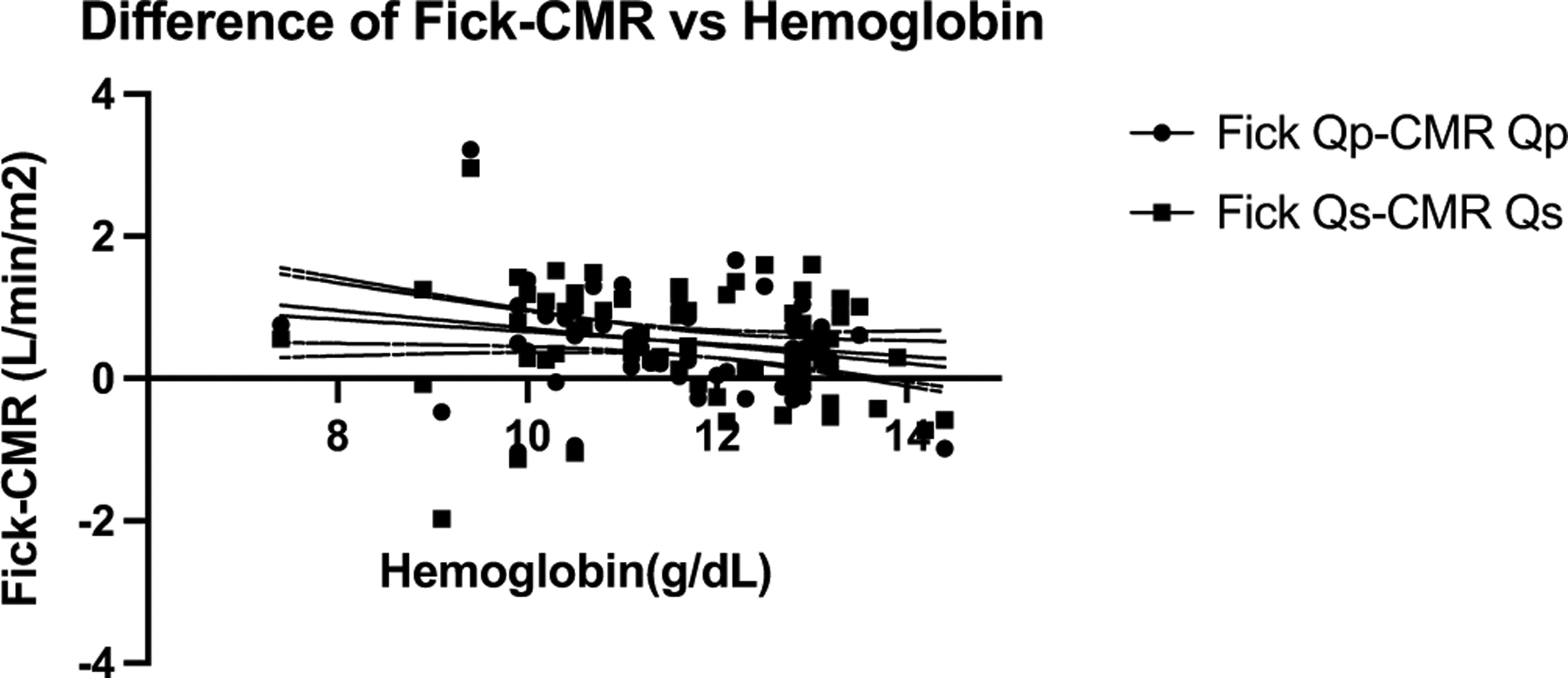
Difference between fick and CMR versus hemoglobin. dots or squares represent individual measurements. solid line is best fit. note, as hemoglobin increased, the difference between fick and CMR decreased for both SBF and PBF.

Accounting for haemoglobin using a mixed model linear regression analysis resulted in significant improvements in the correlation between cardiac magnetic resonance and Fick, particularly with anaemic patients; patient age, body surface area, and heart rate had minimal effect (Fig. 2). A sub-analysis of 9 patients with Fontan circulation was performed, and the presence of previous Fontan had little effect on the Bland–Altman analysis and correlation (bias + 0.5 L/min with 95% limits of agreement of −1.0 – + 2.0 with R^2^ of 0.6 for pulmonary blood flow and −1.1 – + 2.1 and R^2^ of 0.5 for systemic blood flow).

Cardiac magnetic resonance flow measurements were also compared to volumetry measurements of right ventricular cardiac output and left ventricular cardiac output in a sub-analysis of the original interventional cardiac magnetic resonance group from those providing usable volumetry data for both ventricles. In 66 cardiac magnetic resonances from 28 unique patients with no significant valve leak, Lin correlation coefficients of systemic blood flow (from phase contrast) and left ventricular cardiac output were 0.8, and for pulmonary blood flow (from phase contrast) and right ventricular cardiac output was 0.8, demonstrating good agreement between volumetry and flow measurements of cardiac output. For completeness, cardiac output data were compared to Fick and results correlated even less with Lin correlation coefficients of 0.5 for both pulmonary blood flow and systemic blood flow.

Finally, the two tailed interrater reliability for cardiac magnetic resonance pulmonary blood flow and systemic blood flow were 0.91 (95% confidence interval 0.86–0.94) and 0.95 (95% confidence interval 0.92–0.97), respectively, demonstrating excellent interrater agreement.

### Radiation exposure

**Figure 3.**
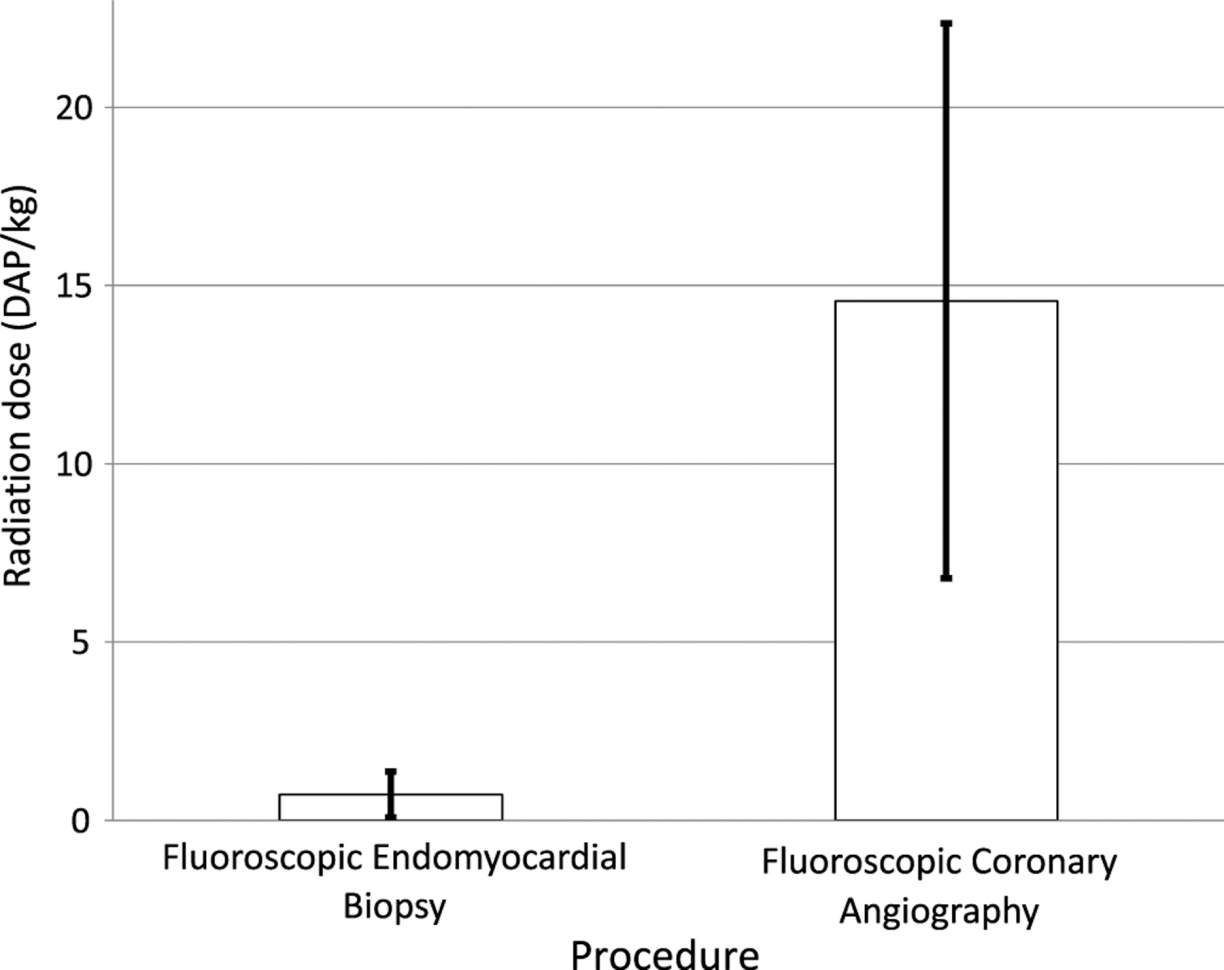
Mean radiation doses for hemodynamics fluoroscopic endomyocardial biopsy and fluoroscopic coronary angiography with units of Gy*m^2^/kg and error bars representing standard deviations (p < 0.05).

The contemporaneous cohort for radiation dose comparison consisted of 60 catheterisations, including 21 patients with 41 haemodynamic and endomyocardial biopsy procedures and 19 patients with 19 complete annual catheterisation procedures, with demographics listed in Table 1. The median ± interquartile range radiation dose for haemodynamics with endomyocardial biopsy (no coronary angiography) was 0.7 ± 0.6 Gy*m^2^/kg and for complete annual catheterisation, 14.6 ± 7.8 Gy*m^2^/kg (Fig. 3). Surveillance coronary angiography represented the dominant contributor to radiation dose during routine surveillance of the orthotopic heart transplant population.

## Discussion

In the paediatric population, cardiac catheterisation is considered the gold standard for post-transplant haemodynamic surveillance, despite its inherent invasiveness and radiation exposure. These data demonstrate that cardiac magnetic resonance measures of flow and cardiac output volumes correlate well with each other, but there is less correlation with Fick. This is likely due to several assumptions required to utilise Fick, including^1^ assumed oxygen consumption,^2^ temporal resolution of data sampling between cardiac magnetic resonance (which takes seconds to measure) and Fick (which relies on samples over a 5–10 minute period). Previous Fontan circulation did not account for the differences in Fick and cardiac magnetic resonance in a sub-analysis. Interestingly, there was a trend with haemoglobin such that the extremes of haemoglobin levels lead to even worse correlation between Fick and cardiac magnetic resonance which could be related to subtle differences in assumed oxygen consumption. cardiac magnetic resonance measures can be limited by flow dispersion from dephasing artifacts such as sternal wires, stents, and other metal implants and signal loss is also possible with turbulent flow leading to error. Direct oximetry and Fick equation, however, are limited by assumed oxygen consumption which come from free breathing, healthy subjects which does not fit a typical paediatric heart transplant catheterisation population. Cardiac magnetic resonance may be, perhaps, the more ideal modality in patients where assumed oxygen consumption and Fick is an even more poor measure. This would include children on beta blockers, those with sick sinus syndrome, and those with mixed pulmonary venous desaturations. Other studies have suggested there is good correlation between these modalities with similar populations, but ours has simultaneous measurements which limits variability due to time and anaesthesia. This work shows there is poor correlation between cardiac magnetic resonance and Fick, albeit on a small population. Further studies to correlate cardiac magnetic resonance measures of volume and flow with outcomes would increase its reliability, especially if Fick could not predict the same outcome.

This study shows the radiation exposure avoided by using interventional cardiac magnetic resonance for haemodynamic catheterisation and endomyocardial biopsy is negligible, as most of the radiation is delivered during coronary angiography. Although interventional cardiac magnetic resonance is an important start, impactful radiation exposure reduction would come from studying transplant related coronary artery vasculopathy with cardiac magnetic resonance. These are important steps in shifting surveillance for transplant rejection and transplant related coronary artery vasculopathy care away from invasive catheterisation toward^1^ non-invasive cardiac magnetic resonance. There is ongoing research to study rejection with cardiac magnetic resonance and interventional cardiac magnetic resonance, but it has been limited in children with fewer financial incentives.^21–23^ Transplant-related cardiac allograft vasculopathy has been studied in children and adults with cardiac CT, myocardial perfusion reserve with cardiac magnetic resonance, and biomarkers including cell-free DNA with limited studies in children.^24–29^ Financial and research investment in studying rejection and transplant-related cardiac allograft vasculopathy is an important step forward to limit ongoing radiation exposure for paediatric heart transplant recipients.

This study is limited in a few ways, including the size of the interventional cardiac magnetic resonance cohort. While large for the field, its size limits the ability to draw firm inferences in some areas including the effect of haemoglobin. There is also a noticeably lower age of children in the haemodynamics with endomyocardial biopsy radiation group because of an increase in transplants in patients under 1 year in recent years. These patients required multiple surveillance catheterisations the first year after transplantation leading to a lower average age of the group.

## Conclusion

Our study shows cardiac magnetic resonance measurements of cardiac output are not concordant and cannot adequately reproduce those measured by invasive catheterisation. There is good correlation, however, between flow and volume measurements in cardiac magnetic resonance suggesting good internal validity. In some patients where the assumptions of the Fick equation are invalid, (e.g. mixed pulmonary venous desaturations, patients on beta blockers or with sick sinus syndrome, extremes of size, etc), cardiac magnetic resonance may correlate better with the patient’s physiology and outcome. Further study of this is needed. While cardiac magnetic resonance can provide catheter guidance and flow measurements without use of radiation, the radiation exposure avoided from a haemodynamic catheterisation alone is too small to be clinically meaningful when compared to the exposure from annual coronary angiography. Future multi-site work must concentrate on these opportunities for accurate diagnosis with cardiac magnetic resonance methods to reduce radiation exposure in this population.

## Figures and Tables

**Table 1.** Patient demographics and anthropomorphics. Column 1 is the demographics of the iCMR vs Fick analysis with means and standard deviations or percentage female. Columns 2 and 3 are the demographics of the radiation cohorts with means and standard deviation or percentage female. P values for the iCMR vs Fick analysis versus the radiation cohort (both groups combined) are given in column 4. Column 5 is the p values for comparison between the two radiation groups

	iCMR vs Fick	Follow up rejection	Complete annual catheterization	P values	P values
Variable	N = 67, 28 patients	N = 41, 23 patients	N = 19, 19 patients	Columns 1 vs 2 + 3	Column 2 vs 3
Age at procedure (years)	12.8 (± 4.8)	10.8 (± 6.2)	13.5 (± 5.7)	0.19	0.20
Age at transplant (years)	5.5 (± 5.7)	7.9 (± 6.8)	8.1 (± 6.5)	<0.01	0.70
Years from transplant to time of procedure	7.3 (± 5.3)	2.0 (± 3.4)	5.0 (± 3.9)	<0.01	<0.01
Female	54%	35%	53%	0.11	0.50
Body Surface Area (m^2^)	1.4 (± 0.4)	1.2 (± 0.5)	1.3(± 0.4)	0.042	0.30
Hemoglobin (g/dL)	11.7 (± 1.5)	–	–	–	–
Estimated VO2 (mL/min/m^2^)	137 (± 14.3)	–	–	–	–
Fluoroscopy Time (minutes)	–	4.6 (± 6.3)	11.2 (± 5.6)	–	<0.01
